# An unusual case of bilateral hydroureteronephrosis caused by uretero-vesico malakoplakia in a young male: a case report and review of the literature

**DOI:** 10.1186/1757-1626-2-7527

**Published:** 2009-05-29

**Authors:** Rashmi Patnayak, Mandyam Kumaraswamy Reddy, Srinivasn Subramanian, Amitabh Jena, Gangi Ravisankar, Rambabu Satya Dandu

**Affiliations:** 1 Department of Pathology, Sri Venketeswar Institute of Medical SciencesTirupati, Andhra Pradesh-517507India; 2 Department of Urology, Sri Venketeswar Institute of Medical SciencesTirupati, Andhra Pradesh-517507India; 3 Department of Surgical Oncology, Sri Venketeswar Institute of Medical SciencesTirupati, Andhra Pradesh-517507India

## Abstract

**Introduction:**

Malakoplakia is an unusual chronic inflammatory disease. Malakoplakia of the bladder and ureter is quite rare.

**Case presentation:**

We present a case of a young male diagnosed as malakoplakia of urinary bladder and ureter. He presented with bilateral hydroureteronephrosis. The patient underwent left ureterocystotomy. Again, he was admitted after a gap of ten years and with features of end stage renal disease.

**Conclusion:**

This rare case of urinary bladder and ureter malakoplakia in a young male is presented, to stress upon the fact that though it is a chronic inflammatory disease, yet its outcome over the years is dismal.

## Introduction

Malakoplakia is an unusual chronic inflammatory disease, which presents as a plaque or a nodule. Malakoplakia can affect all body organs; bladder being the most frequently affected one, particularly in females [1]. Patients of any age may develop malakoplakia, but the peak occurrence is in middle age. The disease is more common in immunocompromised individuals.

We hereby present an unusual case of a young male diagnosed as malakoplakia of urinary bladder and ureter with bilateral hydroureteronephrosis, which progressed to end stage renal disease over a period of ten years highlighting the pathological aspects as histopathology is important in establishing diagnosis.

## Case presentation

An 18-year-old Indian male presented with history of low-grade fever and bilateral pedal edema for about one and half months in 1995. He had no history of urinary symptoms including oliguria or burning micturition. There was no notable finding in physical examination except bilateral pedal edema and facial puffiness. He was a non-smoker and teetotaler. He was not diabetic. He was normotensive. There was no significant past history. Tests for retrovirus; hepatitis B and C were negative. Routine investigation revealed normal hematocrit (Hemoglobin-14 gm/dl) and slightly elevated renal parameters (Serum creatinine-1.6 mg/dl, blood urea-51 mg/dl), apart from albuminuria and pyuria on urine analysis. On further evaluation his 24-hour urine protein was found to be 959 mg/day. Urine culture was sterile. Ultrasound of abdomen and pelvis revealed cholelithiasis and bilateral hydroureteronephrosis and polypoidal mixed echogenic lesions mostly at the base of the bladder. IVU (Intra venous urogram) was not done due to altered renal parameters and patient was subjected to diuretic Renogram to assess functional status and drainage pattern of the kidneys. Renogram revealed decreased tracer uptake and delayed excretion of tracer on both sides. Cortical margin was very much thinned out in left kidney when compared to right kidney. Relative contribution of left kidney was 32% with GFR of 24 ml/min. Micturating cystourethrogram has not revealed any evidence of reflux into upper tracts. His urodynamic evaluation was normal with evidence of normal compliance and normal voiding pressure. Cystoscopy done for the evaluation of polypoidal lesions of the bladder revealed pinkish-yellow nodules of 2 to 3.5 cm size over trigonal area, on left ureteric orifice and at bladder neck. Ureteroscopy was performed on both sides in view of the bilateral hydroureteronephrosis, which revealed multiple white nodules in the lower segment of left ureter. Biopsy of the trigonal and lower ureteric lesions were performed using flexible biopsy forceps.

**Figure 1.**
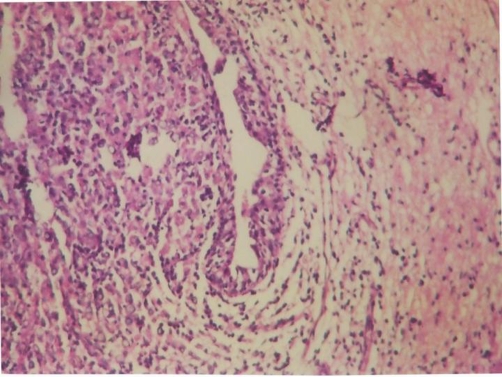
Macrophages (von Hansemann cells) with tiny basophilic inclusions (Michaelis Gutmann bodies) (H&E × 200).

**Figure 2.**
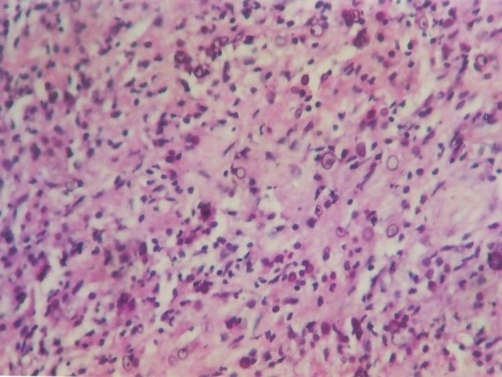
Higher magnification showing intra and extra cellular Michaelis Gutmann bodies (H&E × 400).

**Figure 3.**
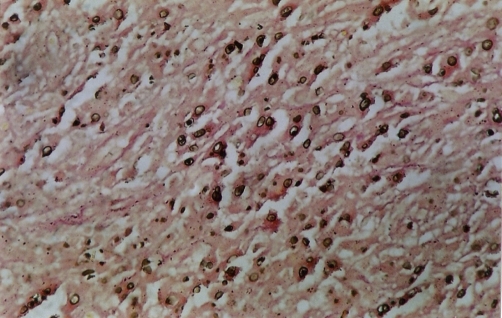
Michaelis Gutmann bodies (Von kossa).

**Figure 4.**
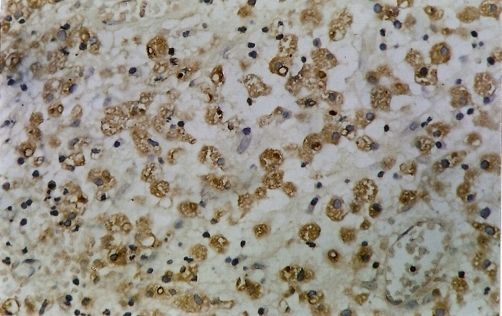
Macrophages showing positivity for CD68.

Microscopy has revealed the presence of sheets of large granular macrophages (von Hansemann cells) with tiny basophilic periodic acid-Schiff and von Kossa positive calcific inclusions (Michaelis Gutmann bodies), admixed with lymphocytes and plasma cells (Figures 1-3). The macrophages were immunohistochemically positive for CD68 antibodies (Figure 4).

**Figure 5.**
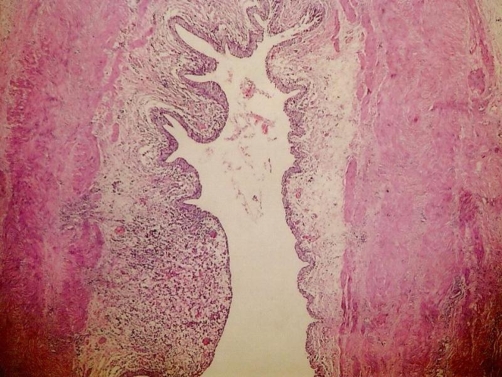
Malakoplakia of left ureter (H&E × 200).

Patient was put on antibiotic treatment and left ureterocystotomy was done to prevent deterioration of existing function of the left kidney. Histopathological examination of excised distal segment of left lower ureter also revealed malakoplakia (Figure 5). He was put on suppressive antibiotics and advised regular follow-up but was lost for follow-up after six months. He turned up again after a gap of ten years with feature of end stage renal disease. According to the patient, he was alright for almost nine years. Then he was evaluated for chronic renal failure and detected to have hypertension in 2004. He was advised to undergo renal transplantation but the patient could not afford it. He was admitted again in 2005 with complaint of low grade intermittent fever with chills and rigor, oliguria, urgency and difficulty in micturition of one month duration. There was history of facial puffiness and rapidly worsening breathlessness of 10days duration. There was also one episode of epistaxis. He had been in altered sensorium for one day prior to the day of admission. At the time of presentation, clinical examination revealed anemia, edema, hypertension, bilateral basal rales and altered sensorium. Investigations revealed severe renal insufficiency (serum creatinine was 19.9 mg/dl and serum urea was 340 mg/dl), hyperphosphatemia, and anemia (Hemoglobin was 5.1 gm/dl), leucocytosis with left shift, thrombocytopenia, pyuria and pulmonary edema. Ultrasound revealed normal sized right kidney with echogenic cortex and loss of corticomedullary distinction, left kidney contracted with increased echoes. He underwent 3 sessions of hemodialysis. During his hospital stay he developed one episode of generalized tonic clonic seizures for which phenytoin was started. Prognosis of patients condition and need for long term regular therapy was explained to the relatives. But they requested to take the patient home. Hence, he was discharged and advised treatment with Cefodoxime, Pantoprazole, Calcium, Multivitamin and Phenytoin. The patient succumbed to the illness soon after.

## Discussion

Malakoplakia was first described in the early 1900s as yellow soft plaques that were seen on the mucosa of the urinary bladder. It is a chronic inflammatory disease that affects the genitourinary tract with a special affinity for bladder. Though there is female preponderance in the genital tract, yet in general, males above the age of 50 years are more affected [1]. The symptoms of bladder malakoplakia are hematuria and signs of urinary tract infection such as hesitancy, dysuria, and frequency. Patients may have variable proteinuria, and also increase number of leukocytes and erythrocytes in their urine.

Nearly 90% of the patients have urine infections by coliforms and 40% have autoimmune disease or some type of immunodeficiency [1]. *E coli* are the most common gram-negative bacteria isolated.

The exact pathogenesis is unknown, but it is generally thought to be result of chronic bacterial infections in patients with chronic debility or immunosupression. According to Curran the pathogenesis of malakoplakia mainly involves impaired host defenses and defective phagocytosis. Defective macrophage killing of bacteria results in an accumulation of bacterial degradation products and a granulomatous reaction, which clinically manifests as the formation of a papule, a plaque, or ulceration [2]. Grignon and Sakr are of the opinion that the bacteria ingested by the macrophages are destroyed but not completely digested. They persist in the phagolysosomes and become mineralized; resulting in the pathognomonic calcified intracellular inclusions, the Michaelis-Gutmann bodies [3]. Definite diagnosis is made by biopsy only as the cystoscopic and imaging appearances of malakoplakia are nonspecific and mimic those of neoplasms.

Microscopically, there are collections of histiocytes with granular acidophilic cytoplasm (von Hansemann cells) admixed with Michaelis-Gutmann bodies and infiltrated by varying numbers of lymphocytes and plasma cells. Michaelis-Gutmann bodies are pathognomonic of malakoplakia. Gram stain may demonstrate gram-negative bacteria. Immunohistochemical studies demonstrate positive results for CD68 antibodies, lysosomes, and α-chymotrypsin in the macrophages.

Histopathologically the lesions of Xanthogranulomatous cystitis closely resemble malakoplakia, but it lacks Michaelis-Gutmann bodies [4].

The initial treatment of malakoplakia consists of prompt treatment of urinary infection and surgery for the affected site. Usually malakoplakia is a self limiting benign condition associated with good prognosis. Very few cases of acute renal failure by multifocal malakoplakia are reported in literature [5].

## Conclusion

We present this case of malakoplakia of urinary bladder and ureters in a young immunocompetent male, as most of the cases described in the literature are adults with associated immunodeficiency conditions; and further bilateral hydroureteronephrosis due to malakoplakia is quite uncommon. In addition, though malakoplakia is a chronic inflammatory disease yet this particular case developed severe renal insufficiency over a period of years. Hence, we would like to stress upon the fact that lesions of malakoplakia though innocuous, should be closely followed up and the patients should be regularly monitored to prevent development of renal insufficiency at a later date.

## List of abbreviations

GFR: Glomerular filtration rate
IVU: Intra venous urogram
